# Radiation Recall Pneumonitis Induced by Anti-PD-1 Blockade: A Case Report and Review of the Literature

**DOI:** 10.3389/fonc.2020.00561

**Published:** 2020-04-28

**Authors:** Yu Chen, Zhaoqin Huang, Ligang Xing, Xiangjiao Meng, Jinming Yu

**Affiliations:** ^1^Cheeloo College of Medicine, Shandong University, Jinan, China; ^2^Department of Radiation Oncology, Shandong Cancer Hospital and Institute, Shandong First Medical University and Shandong Academy of Medical Sciences, Jinan, China; ^3^Department of Radiology, Shandong Provincial Hospital Affiliated to Shandong First Medical University, Jinan, China

**Keywords:** radiation recall pneumonitis (RRP), anti-PD-1 blockade, thoracic radiation, immunotherapy, Camrelizumab

## Abstract

**Background:** Radiation recall pneumonitis (RRP) is an unpredictable but relatively severe subclinical radiation damage which occurs in the previously irradiated fields of pulmonary tissue after administration of a systemic agent. Previous reports of RRP were mainly attributed to chemotherapy and molecular-target agents. RRP induced by immunotherapy has been rarely reported. Here we describe a case of a novel pattern of RRP induced by anti-PD-1 blockade Camrelizumab 2 years after radiotherapy, with some focus on further understanding of this phenomenon.

**Case Report:** A 64-year-old man with non-small cell lung cancer (NSCLC) received two cycles of chemotherapy with cisplatin and pemetrexed first. Subsequently, he underwent concomitant chemoradiotherapy with cisplatin and pemetrexed to simultaneous integrated boost (SIB) radiotherapy. After 15 months, due to tumor progression and brain metastasis, he started with administration of anti-PD-1 blockade Camrelizumab (200 mg q2w) and stereotactic radiosurgery (SRS). The patient developed fever, dyspnea and cough after the eighth administration of Camrelizumab. Meanwhile, his chest CT revealed patchy consolidation and ground-glass opacities localized within the previously irradiated area. Subsequent treatment regimen was adjusted to 80 mg q12h prednisolone with discontinuation of Camrelizumab. Then the symptoms gradually eased and reexamination of CT showed significant improvement in RRP after 2 weeks.

**Conclusion:** Our case report presents a novel pattern of RRP induced by anti-PD-1 blockade Camrelizumab 2 years after radiotherapy. This indicates that previous radiotherapy combined with subsequent anti-PD-1 blockade has a potential to cause overlapping damage to lung, suggesting that intensive attention might be needed for patients who are treated with anti-PD-1 blockade in conjunction with a prior history of thoracic radiation.

## Background

Radiation recall pneumonitis (RRP) is an unpredictable, poorly understood phenomenon which occurs in the previously irradiated fields of pulmonary tissue after subsequent administration of a pharmacological agent. Typically, radiation-induced inflammatory reactions happen within 6–9 months following radiotherapy, whereas RRP usually occurs after a longer non-inflammatory interval and only after the application of systemic anti-tumor agents. To our knowledge, several studies reported that anti-tumor drugs could cause RRP with the majority attributed to chemotherapy and molecular-target agents (1–4). RRP induced by immunotherapy has been rarely reported.

Lung toxicity is one of the most common side effects of both thoracic radiation and immunotherapy (5, 6). Therefore, the combination of radiotherapy with immunotherapy has a potential to cause overlapping damage to the lung. Though with significantly improved progression-free survival (PFS) and overall survival (OS), the KEYNOTE-001 trial demonstrated that there was a statistical difference in treatment related pulmonary toxicities between patients with advanced NSCLC who had been treated with any RT before receiving pembrolizumab than those who had not (13 vs. 1%, *p* = 0.046) (7). Likewise, the PACIFIC study showed that pulmonary toxicities of any grade were more common in the durvalumab arm than the placebo arm (33.9 vs. 24.8%, respectively) (8). The available data above indicate that patients receiving anti-PD-1/PD-L1 blockades with a history of any RT are prone to develop lung toxicities. Our case presents a novel pattern of the potential overlapping damage with a relatively long interval between the end of radiotherapy and the initiation of immunotherapy, which is defined as RRP. Currently, with the approval of immunotherapy for clinical use, a growing body of patients are treated with anti-PD-1/PD-L1 blockades in addition to previous exposure to thoracic radiation. Hence, a comprehensive understanding of RRP induced by immunotherapy is particularly crucial.

Up to now, due to limited data, the pathophysiological mechanism remains unclear. And there is no explicit consensus with regard to the specific guidelines for diagnosis and treatment of RRP. In our review, we describe a case of a novel pattern of RRP induced by anti-PD-1 blockade Camrelizumab 2 years after radiotherapy, with some focus on the potential mechanism, predictive risk factors and appropriate management of RRP.

## Case Presentation

A 64-year-old non-smoking man, with an Eastern Cooperative Oncology Group Score (ECOG) of 1, was admitted to our hospital, suffering from a cough and expectoration with blood in sputum. The enhanced CT revealed a 2.3 × 2.2 cm mass in the left hilar area with invasion of the mediastinum. In addition, the CT scan also exposed multiple enlarged lymph nodes in mediastinum and supraclavicular region. A bronchoscopy was then performed and the pathology results showed poorly differentiated carcinoma (at the opening of the lower lobe of the left lung), which, combined with immune markers, was diagnosed as adenocarcinoma. The primary stage was cT4N3M0 with no mutations found in epidermal growth factor receptor (EGFR) gene, anaplastic lymphoma kinase (ALK) gene, and ROS1 gene.

**Figure 1:**
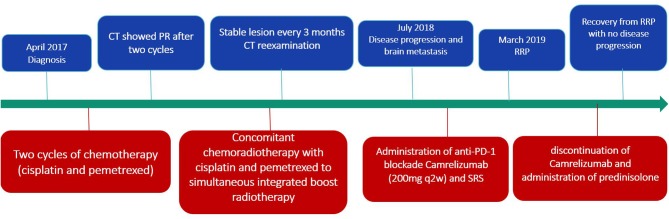
Timeline of disease status and corresponding treatment regimens.

For fear of radiation pneumonitis, the patient initially refused radiotherapy. Therefore, the treatment plan was chemotherapy (cisplatin 40 mg day 1–2, 50 mg day 3; pemetrexed 1 g day 1) first (Figure 1). A CT scan showed that he achieved partial response after two cycles. At this point, the patient agreed to receive radiotherapy. Subsequently, the treatment plan was adjusted to concomitant chemoradiotherapy with cisplatin and pemetrexed to simultaneous integrated boost (SIB) radiotherapy. The specific RT dose is planning target volume (PTV) 58.0 Gy in 29 fractions (2.0 Gy^*^29) with SIB to a total dose of 63.8 Gy in 29 fractions (2.2 Gy^*^29). The volume of lung receiving 20 Gy (V20) was 17% and the mean lung dose (MLD) was 13.5 Gy. During chemoradiotherapy, the patient suffered from grade 3 leukopenia accompanied with grade 1 loss of appetite and no other adverse effects occurred. Reexamination every 3 months showed that the lesion was stable.

**Figure 2:**
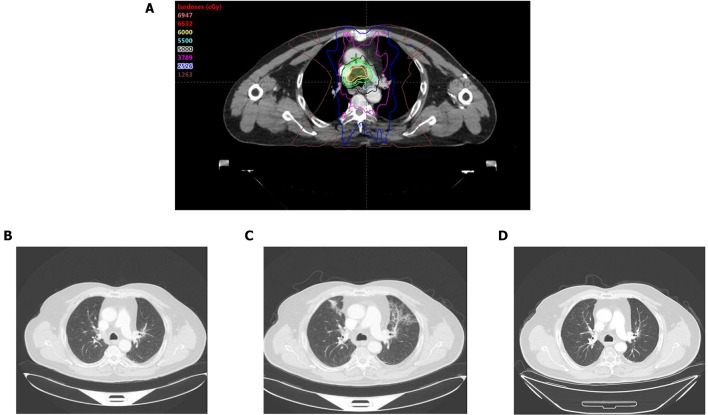
**(A)** Radiation field. **(B)** Before administration of Camrelizumab, CT scan showed no pneumonitis manifestation. **(C)** After administration of Camrelizumab, CT scan revealed patchy consolidation and ground-glass opacities localized within the previously irradiated area. **(D)** Reexamination of CT showed significant improvement in pneumonitis 2 weeks after administration of prednisolone.

Fifteen months later, the brain MRI revealed two enhancement foci in the left frontal lobe, considering the great possibility of metastatic tumors. The chest CT showed slight enlargement in the original lesion of lung with a 2.7 × 2.3 cm mass. The following treatment started with administration of anti-PD-1 blockade Camrelizumab (200 mg q2w) and SRS with a dose regimen of 50 Gy in 10 fractions for brain metastatic lesions. The regular reexamination of chest CT and brain MRI showed that the patient achieved partial response with promising efficacy evaluation. However, after the eighth administration of Camrelizumab, the patient developed fever, dyspnea and cough. The fever lasted for 3 days with the peak of 39°C. Blood oxygen saturation fluctuates between 92 and 96% in the nasal duct oxygen state. Paroxysmal coughs are accompanied by small amounts of white phlegm. Meanwhile, his chest CT revealed patchy consolidation and ground-glass opacities localized within the previously irradiated area (Figure 2). However, there was no significant change from the last results in his brain MRI. A bronchoscopy and biopsy were recommended to clarify the nature of the lesion, but the patient refused to undergo invasive testing. At this time, blood culture and sputum culture examinations did not present any significant infection. The analysis of CT scan lesion characteristics and the shift of circulating tumor cells (CTCs) from positive to negative in blood samples suggested that the emerging lesions are less likely to be tumor progression. Based on the facts above, the patient was suspected with RRP induced by Camrelizumab. In addition to the immediate discontinuation of Camrelizumab, the subsequent treatment regimen was adjusted to 80 mg q12h prednisolone with following stepwise reduction over weeks (80 mg q12h 3 days; 60 mg q12h 3 days; 40 mg q12h 3 days; 30 mg q12h 3 days; 20 mg q12h 3 days; 10 mg q12h 3 days). Then the symptoms gradually eased and reexamination of CT showed significant improvement in pneumonitis after 2 weeks, which further confirmed the diagnosis of RRP induced by Camrelizumab. Notably, during the withdrawal of Camrelizumab, the primary lung cancer lesions did not change significantly compared with the previous ones, indicating that the anti-tumor effect of Camrelizumab persisted to some extent.

## Discussion

Radiation recall pneumonitis is regarded as a type of radiation-related lung damage that manifests as acute inflammation lymphocytes infiltration confined to previously irradiated lung tissue, weeks, months, or even years after the end of radiotherapy in response to a sequential systemic agent stimulation. After summarizing the previously reported cases of RRP, we observe that the clinical manifestation varied from completely asymptomatic to severely symptomatic with non-productive cough and dyspnea on exertion (1–4). But one common feature all patients presented was that CT showed confluent ground glass opacities corresponding closely to their prior radiotherapy fields. The occurrence of RRP is drug-specific for any individual patient, and now it is almost impossible to predict which patients will respond to which drugs.

Camrelizumab (SHR-1210) is a novel humanized PD-1 monoclonal antibody that can be potentially applied to the treatment of solid tumors (9, 10). Upon administration, Camrelizumab blocks the binding of PD-1 to its ligands PD-L1, thus restoring and enhancing immune function through the activation of cytotoxic T lymphocytes (CTLs) and cell-mediated immune responses against tumor cells. More detailed information of it is being assessed in plenty of clinical trials (10, 11).

In our case, because it has been 2 years since the completion of radiotherapy, it seems to be unlikely to be the conventional radiation pneumonitis. As is well-known, immunotherapy has great potential to cause pneumonitis as well. Based on currently available data, the incidence of the immune-related pneumonitis after anti-PD-1/PD-L1 blockades treatment varies from 1 to 10% (12). Generally, the distribution of immune-related pneumonitis is not limited to a specific area of the lung. A study conducted by Voong et al. provided an in-depth understanding of immune-related pneumonitis and further compared with radiation-related pneumonitis in patients treated with prior thoracic radiation (13). It demonstrated that there were spatial distribution differences between the two types of pneumonitis with immune-related pneumonitis mainly within lung volume that is outside the high radiation dose delivery. Therefore, though the patient in our case developed fever, dyspnea and cough after the eighth administration of Camrelizumab, CT scan showed that the ground glass opacities with consolidations mainly distributed within the previously irradiated area, which was not completely corresponding to immune-related pneumonitis. Because the patient in our case refused to undergo bronchoscopy and biopsy, pathological characteristics were unavailable. Nonetheless, given the current research on radiation-related pneumonitis and immune-related pneumonitis, we can find that both types of the pneumonitis are triggered by the body's potent immune response and meanwhile are accompanied by a large accumulation of lymphocytes (14, 15). Therefore, it may be hard to differentiate radiation-related pneumonitis and immune-related pneumonitis by pathological characteristics. Further examination including the blood and sputum culture did not present any significant infection with a distinct shift of CTCs from positive to negative in blood samples. Besides, the symptoms gradually eased and reexamination of the CT showed significant improvement after discontinuation of Camrelizumab and administration of prednisolone. Hence, based on the evidence above, it can be concluded that the pneumonitis was RRP induced by Camrelizumab combined with or without immune pneumonitis.

To our knowledge, several studies have reported that there are anti-tumor drugs which can cause RRP. Togashi et al. reported a case of a lung cancer patient suffering from RRP induced by erlotinib with relatively high plasma concentration after 7 months of radiotherapy (1). Schwarte et al. presented a patient with metastasized esophageal carcinoma developed RRP induced by gemcitabine after 8 months of radiotherapy (2). Other different kinds of pharmacologic anti-tumor agents have also been involved in this inflammatory reaction as taxanes, paclitaxel (3, 4). RRP induced by immunotherapy has been rarely reported. To the best of our knowledge, up to now, there are only two previous studies reporting RRP induced by immunotherapy. The study conducted by Shibaki et al. reported two cases of RRP induced by nivolumab in which the interval between the end of radiation and the diagnosis of RRP were 660 and 664 days, respectively (16). Another case reported by McCusker et al. showed that a patient with malignant pleural mesothelioma developed life-threatening RRP after a single infusion of nivolumab with a 7-months interval after the completion of proton beam therapy (17). Of note, an FDA approval summary of all pembrolizumab studies shows that pneumonitis is more likely to happen in patients with a history of prior thoracic radiation compared with those without that exposure (6 vs. 2.6%), potentially indicating the existence of RRP induced by pembrolizumab (18).

The specific biological mechanism of RRP is unclear. The latent radiation effects in the previously irradiated tissue are possibly evoked by anti-PD-1 blockade. One hypothesis indicates that administration of systemic anti-tumor agents after radiotherapy triggers a “remembered” reaction in the remaining surviving cells within the previously irradiated areas (19). Notably, the underlying “remembered” reaction may be the result of immunomodulatory effects of previous radiotherapy, which allows the remaining surviving cells to overreact to anti-PD-1 blockade, thus leading to inflammation. Evidence shows that a variety of immunomodulatory effects might be involved in this process. First, it has been demonstrated that radiotherapy can upregulate the expression level of immunogenic cell surface markers such as ICAM-1, MHC-1, and Fas, which makes the cells more susceptible to CD8+ T cell recognition and destruction (20, 21). Second, radiotherapy could enhance antigenicity and sensitivity of the remaining cells. After treated with anti-PD-1 blockade, these cells are prone to release damage-associated molecular pattern (DAMP) especially double stranded DNA, which further activate innate and adaptive immune responses through cyclic GMP-AMP synthase (cGAS)- stimulator of interferon genes (STING) pathway and induce the expression of certain cytokines that are involved in inflammatory response, such as type I interferon, interleukin-1(IL-1), interleukin-6(IL-6), tumor necrosis factor α (TNFα), and transforming growth factor β (TGFβ) (19, 22–25). Third, studies have shown that radiotherapy appears to facilitate T cell recruitment to irradiated areas by promoting the release of chemokines as CXCL16 (26). The increased T cells within the irradiated areas lowers the inflammatory response threshold, thus increasing the likelihood of developing RRP after administration of anti-PD-1 blockade. Last but not least, there is evidence which illuminates that radiotherapy plays an unexpected part in the upregulation of PD-L1 expression, which might lead to the irradiated lung tissue hypersensitivity and overreaction to anti-PD-1 blockades (27, 28). It seems that the “remembered” reaction of previously irradiated lung tissue is mainly attributed to the enhanced sensitivity to systemically administrated anti-PD-1 blockade. Except for the “remembered” reaction hypothesis of radiotherapy, another hypothesis suggests that radiation leads to heritable mutations within surviving cells, which further produce a subgroup of defective stem cells that are sensitive to anti-tumor drugs (19). The third hypothesis indicates the pharmacokinetics of the systemic anti-tumor agents is altered by local vascular permeability or proliferative changes induced by radiotherapy (29). To date, there is a lack of experimental and clinical evidence on the RRP that can definitely support any possible hypothesis. Further investigation is required and more efforts need to be taken.

With the increasing availability and expanding use of anti-PD-1/PD-L1 blockades, to minimize the incidence of RRP induced by them, it is of great importance to identify certain predictive risk factors of RRP. For one thing, it has been found that some dosimetric factors including V20 and MLD have a close correlation with the incidence and severity of radiation pneumonitis (RP). A study conducted by Tsujino et al. (30) shows that a V20 <25% corresponds to a lower incidence of RP whereas a V20 > 30% is markedly associated with a higher incidence of grade 2 or greater RP. Besides, Luna et al. evaluated 302 locally advanced NSCLC patients with definitive chemoradiation to a median dose of 66.6 Gy in 1.8 Gy daily fractions. Both the univariate analysis and multivariate analysis emphasized the importance of lung V20 (16.4%) and MLD (15.7%) as potential predictors of RP (31). Based on the fact above, we speculate that the assessment of V20 and MLD of previous radiotherapy might play a significant part in estimating the risk of RRP to some extent. Future studies are warranted to further investigate. For another, the time interval between the end of radiotherapy and initiation of systemic agents might have a considerable effect on the development of RRP (32). A study conducted by Chiang et al. demonstrated that patients in whom epidermal growth factor receptor-tyrosine kinase inhibitor (EGFR-TKI) was administered within 90 days after the end of radiotherapy had higher incidence rates of RRP than those of patients who initiated EGFR-TKI treatment after 90 days (21 vs. 2.1%, *p* = 0.005) (33). Of particular interest, study has shown that sex, age, performance status, smoking history, preexisting ILD, baseline pulmonary function might be not associated with RRP (33). Due to limited data, the possible predictive risk factors above remain to be further confirmed.

At present, there is no clear consensus on specific guidelines for diagnosis and treatment of RRP. RRP is generally diagnosed through the evaluation of treatment history, clinical symptoms, physical examination and radiologic images. Typically, patients with RRP might develop fever, cough, and dyspnea after administration of systemic anti-tumor agents with a prior history of radiotherapy. The radiologic images show confluent ground glass opacities corresponding closely to their previous irradiated fields. Invasive biopsy is an important auxiliary diagnostic basis, but not normally indispensable. Blood culture and blood sample examinations help distinguish RRP from infections and disease progression. In summary, caution is needed when anti-PD-1/PD-L1 blockades are administered to patients who have undergone previous radiotherapy. Clinicians should pay more attention to symptoms of patients such as cough, fever and inflammatory imaging manifestation, which can achieve early detection, and treatment.

Currently, the accepted standard treatment for RRP includes discontinuation of the systemic anti-tumor agents, administration of corticosteroids, and supportive medical care (3). Prednisolone is one of the most commonly used corticosteroids and could improve symptoms of RRP and minimize lung tissue toxicity. In our case, the patients showed durable tumor control after discontinuation of Camrelizumab. RRP achieved improvement after initiation of prednisolone with 80 mg q12h. For fear of recurrence, the patient did not continue to use Camrelizumab after recovery from RRP. Of particular concern, due to limited data, it remains to be further studied whether continued use of anti-PD-1 blockade after the control of RRP causes its recurrence.

## Conclusion

In conclusion, we reported a case of novel pattern of RRP induced by anti-PD-1 blockade Camrelizumab 2 years after radiotherapy. To our best knowledge, this is the third study describing RRP induced by anti-PD-1 blockade with the previous two related to nivolumab. Though with a relatively long time interval, previous radiotherapy combined with subsequent anti-PD-1 blockade still has a great potential to cause overlapping damage to lung. Currently, the specific pathophysiological mechanism of RRP still remains unclear. Of note, the “remembered” reaction in the remaining surviving cells within the previously irradiated areas caused by the immunomodulatory effects of radiotherapy might be the most persuasive hypothesis. Risk for RRP should be kept in mind all the time while initiating anti-PD-1 blockade treatment in patients with a prior history of thoracic radiation. Once grade 3–5 RRP occurs, discontinuation of the administered agent and application of systematic steroids are a feasible choice for treatment.

In clinical practice, with the widespread use of anti-PD-1 blockades, an increasing number of patients are treated with them in addition to having previously been exposed to thoracic radiation. Therefore, it is quite significant to study deeply to figure out the specific mechanism and predictive risk factors of RRP. A further analysis of the specific guidelines for diagnosis and treatment regimen also has important clinical significance and more efforts should be taken to demonstrate.

## Data Availability Statement

All data used in this case report are included in this article.

## Ethics Statement

This case report was approved by the local ethical committee. Informed and written consent was obtained from the patient to use his clinical information and data.

## Author Contributions

XM designed the study, edited, and approved final manuscript. JY made critical appraisal and approved final manuscript. ZH and LX collected the data and made some radiological analysis. YC analyzed the data and drafted the article.

## Conflict of Interest

The authors declare that the research was conducted in the absence of any commercial or financial relationships that could be construed as a potential conflict of interest.

## Abbreviations

RRP: radiation recall pneumonitis
PD-1: programmed death-1
PD-L1: programmed death ligand-1
NSCLC: non-small cell lung cancer
SRS: stereotactic radiosurgery
PFS: progression-free survival
OS: overall survival
EGFR: epidermal growth factor receptor
ALK: anaplastic lymphoma kinase
SIB: simultaneous integrated boost
PTV: planning target volume
V20: volume of lung receiving 20 Gy
MLD: mean lung dose
CTCs: circulating tumor cells
CTLs: cytotoxic T lymphocytes
TKI: tyrosine kinase inhibitor
IL-1: interleukin-1
TNFα: tumor necrosis factor α
TGFβ: transforming growth factor β
DAMP: damage-associated molecular pattern
cGAS: cyclic GMP-AMP synthase
STING: stimulator of interferon genes
RP: radiation pneumonitis.
